# Surface melt driven summer diurnal and winter multi-day stick-slip motion and till sedimentology

**DOI:** 10.1038/s41467-019-09547-6

**Published:** 2019-04-08

**Authors:** Jane K. Hart, Kirk Martinez, Philip J. Basford, Alexander I. Clayton, Benjamin A. Robson, David S. Young

**Affiliations:** 1 Geography and Environment, University of Southampton, Southampton, SO17 1BJ UK; 2 Electronics and Computer Science, University of Southampton, Southampton, SO17 1BJ UK; 3 Winchester College, College St, Winchester, SO23 9NA UK; 4 Department of Geography, University of Bergen, Bergen, 5020 Norway

## Abstract

Fluctuations in glacier motion are very common and are thought to be controlled by subglacial hydrology and till deformation. There are few instrumented studies that have monitored seasonal changes. We use the innovative Glacsweb subglacial in situ wireless probes, combined with dGPS and custom geophone data from an Icelandic soft-bedded temperate glacier, to show that there are two distinct seasonal styles of speed-up events. Relatively small diurnal events occur during the melt season, whilst during winter there are larger multi-day events related to positive degree days. These events are accompanied by a distinct pattern of till deformation and basal icequakes. We argue these reflect stick-slip motion which occurs when the glacier hydrological system is unable to accommodate the melt water flux generated by surface melt episodes. We show a rare fully instrumented coupled glacier/till record of contrasting summer and winter stick-slip motion and discuss its implication for till sedimentology.

## Abstract (web summary)

Glacier meltwater can penetrate the glacier bed and act as a lubricant, accelerating retreat. Here, the authors use the unique Glacsweb wireless probe at Skálafellsjökull in Iceland and find evidence for two types of stick-slip events: small diurnal events in summer and large multiday events in winter.

## Introduction

The response of glaciers to climate change, and their contribution to sea level rise, is partly controlled by a combination of subglacial hydrology and sediment deformation^1–3^. Surface melting generates melt water, which may travel through the glacier to its base, where it can regulate glacier velocity via sliding and/or till deformation. Glacier flow is rarely constant, and periodic fast ice flow is common in glaciers on different scales. These can range from short inter-annular speed-up events^4–7^ to glacier surging (where glacier surge speeds can be 10–10,000 times faster than normal flow)^8,9^ to ice stream stagnation/activation^10^. Some speed-up events are due to stick-slip motion. This type of motion is similar to that on earthquake faults, characterised by long periods of slow or no movement (stick phase) (interseismic period of elastic strain accumulation)^11^ interrupted by episodic bursts of movement (slip phase) where glaciers can dramatically increase their velocity over a short, often diurnal time span (minutes to hours)^2,12–15^. The slip phase is often related to sliding or deformation of the till^16^ and is accompanied by a repeated set of basal icequakes.

All types of stick-slip motion are the result of a balance between forces that load an interface and an evolving interfacial strength^3,11,17–19^. It has been argued that a necessary condition for stick-slip cycles are a reduction in frictional strength with increasing sliding velocity after sufficient sliding has eliminated the memory of the sliding history^18^. These models use rate-and-state friction^20^ to describe basal shear stress at the ice-till interface and suggest that basal strength is a decreasing function of water pressure. The model is in agreement with laboratory experiments on subglacial materials^15,19,21–24^, and with glaciological observations from both mountain glaciers and ice sheets^13,14,16^. It has been argued that in Antarctica stick-slip motion is tidally modulated (and this modulation is just a perturbation to a system that would have stick-slip cycles even in the absence of tides);^16,18,25^ whilst in mountain glaciers the causes include variable melt water input and changes in debris load^19,26^.

A key component of glacier motion is the subglacial hydrology system. Those associated with rigid bedrock systems are normally dominated by conduits and linked cavities^27^, while soft bedrock systems are dominated by anastomosing broad flat channels, macroporous water sheets at the ice-bed interface and water within the till^1,28–30^. These later systems will be characterised by higher water pressures than the channel networks and higher velocities^31^.

The behaviour of till is also a vital component of variable glacier motion. Till is a granular material with complex mechanical behaviour. Initial research led to a debate between purely linear viscous models^32^ compared with plastic behaviour^33^. However, more recently various researchers have used a range of analytical techniques (in situ data, sedimentology, geotechnical experiments, computational modelling) to suggest that there may be different behaviours related to effective pressure^3,34–37^. These authors argue that at high effective pressures there is little movement (stick), at intermediate effective pressures there is creep (deformation), and at low effective pressures there is failure (slip). During the creep phase the particles become reorganized in response to increased shear stress, and the till undergoes simple shear within a shear zone^38^.

Numerous researchers^32,35,39^ have shown that when a wet granular material is sheared, this leads to dilation, which will lead to an increase in pore volume. If water is able to flow into the dilating material, this leads to looser packing and a weakening of the sediment^21^, but if water is unable to flow into these spaces, this will result in a drop in water pressure and an increase in strength (dilation strengthening)^22^. Both of these states have been observed in laboratory tests in till^21,40^ as well as from in situ studies beneath modern glaciers^37,41,42^.

Most subglacial environments comprise a mosaic of different bed strengths, ranging from rigid (e.g. bedrock, frozen till, low porosity till, lake sediments, outwash sand and gravels) (‘sticky spots’)^43^ through to deformable till^44^. It has been suggested that these sticky spots will behave as asperities within the stick-slip system^14^.

Detailed measurements of multiple aspects of the glacial system (till deformation, ice velocity, icequakes, etc.) are rare because of the logistical difficulties of monitoring such harsh environments. We present a unique dataset to contrast two distinct styles of stick-slip behaviour during the melt season (diurnal) and winter (multi-day), which are controlled by surface melt-driven discharge. These events lead to cyclic behaviour in the ice and the till. Initially the increase in melt water leads to basal sliding (slip), followed by till deformation and icequakes as the glacier reconnects with the bed. This is followed by a period of low or no deformation (stick) as melt water levels subside, and then reactivation/increase in till deformation and icequakes as the melt water levels build up again and the glacier begins to accelerate. We are able for the first time to quantify the duration of each stage and associated subglacial processes throughout the season based on instrumented data, and relate this to till sedimentology.

## Results

### Wireless environmental sensor network data

**Fig. 1:**
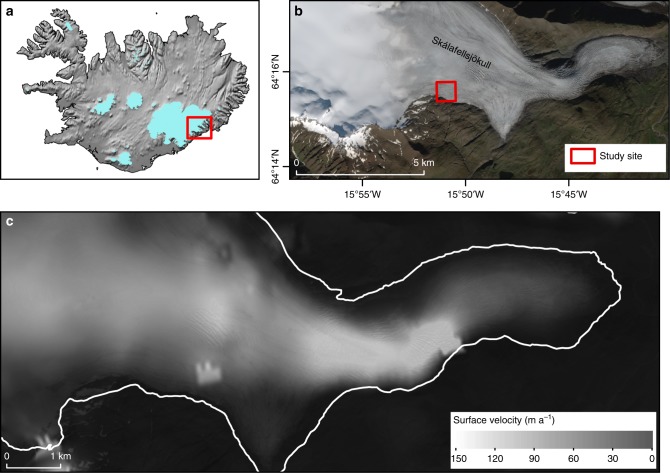
Site map of Skálafellsjökull: **a** location in Iceland (shown by a box) (background: hillshade model based on the national DEM of Iceland); **b** details of the glacier (background: Sentinel 2 natural colour composite from 30 August 2017); **c** glacier wide surface ice velocity estimated from intensity tracking of two Single Look Complex (SLC) TerraSAR-X images with an eleven-day temporal baseline (5th and 16th October 2012) (glacier outline shown in white)

**Fig. 2:**
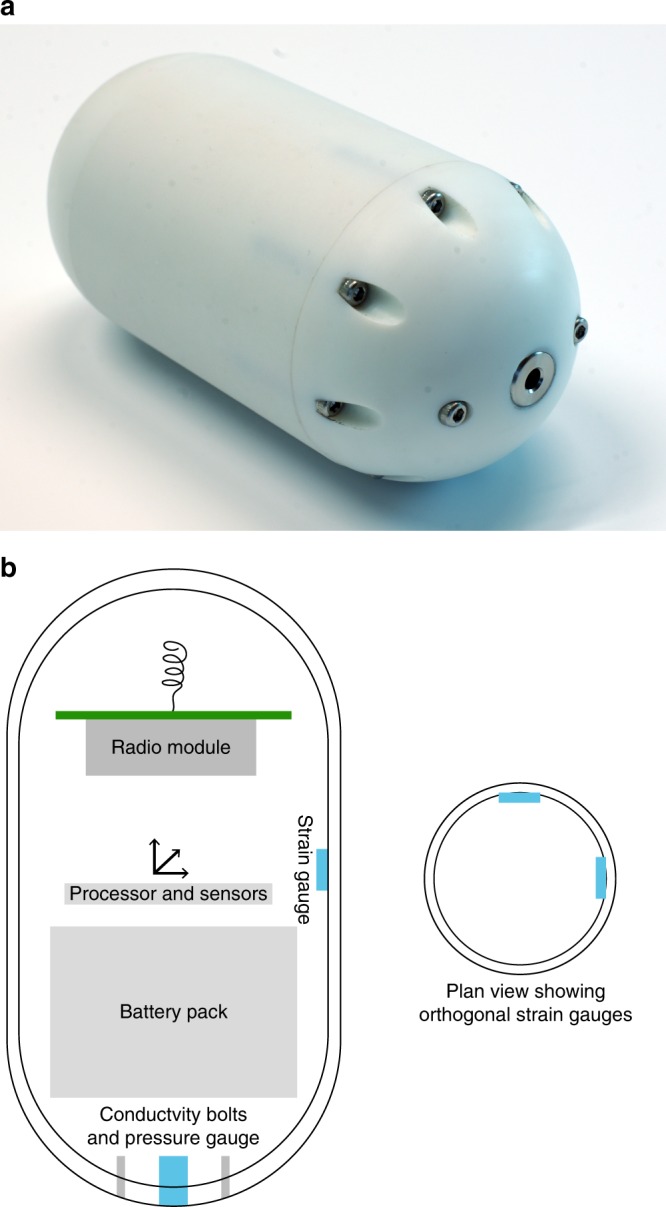
Glacsweb wireless probe details: **a** photograph of the 2012 probe; **b** probe schematic (dimensions: length 0.16 m, width 0.06 m)

We present a dataset collected from a custom wireless Environmental Sensor Network^45^ installed at Skálafellsjökull (Fig. 1), a temperate outlet glacier of the Vatnajökull ice cap^44^. This includes wireless Glacsweb probes (0.16 m long, axial ratio 2.9:1) embedded in the till recording water pressure, case stress, and tilt (Fig. 2). Water pressure was converted to hydraulic head (m) and then compared with the water column height corresponding to local ice flotation from known glacier thickness (flotation pressure—%). Case stress (kPa) (i.e. the force applied to the probes per unit area) was measured by strain gauges that measured the relative compression and extension of the probe case in two perpendicular planes.

Accelerometers measured roll and pitch, from which we calculated tilt. The probe is normally almost vertical during deployment and generally inclines towards the horizontal as the glacier moves over the till. This results in the tilt data changing in one general direction. When the top of the probe moves further in the direction of flow than the bottom, we define this as synthetic. We define movement in the opposite direction as antithetic.

In addition, we used a custom borehole geophone to record icequakes^46^, we measured surface velocities (both horizontal and vertical) with dGPS (from which we were able to calculate bed separation), we determined a glacier-wide velocity from satellite data (using speckle tracking), and we calculated surface melt from the positive degree day algorithm (see Methods for details of these techniques).

**Fig. 3:**
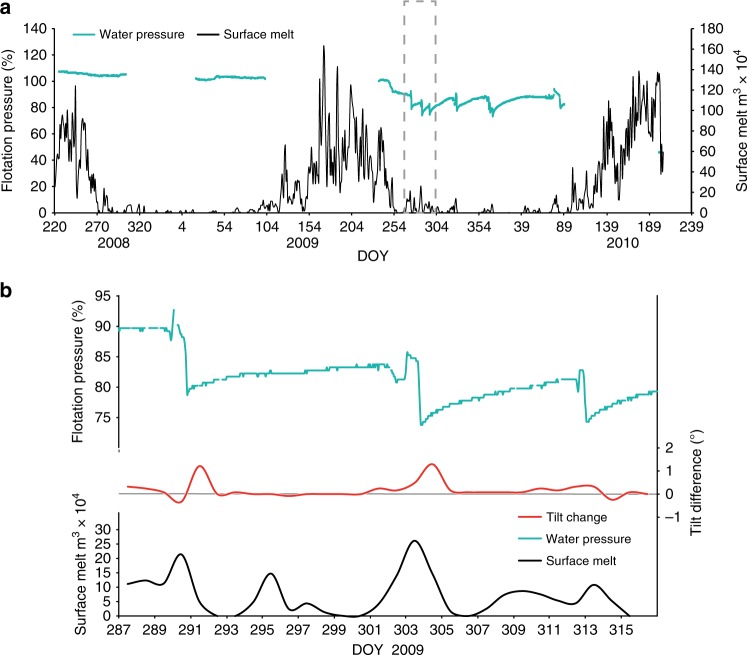
Graphs showing the data from 2008 to 2010: **a** water pressure within the till and surface melt, 2008–2010 (the grey dashed box is enlarged in **b**); **b** detail of water pressure, surface melt and change in tilt during two water pressure cycles day of year (DOY) 287–315 (2009)

**Fig. 4:**
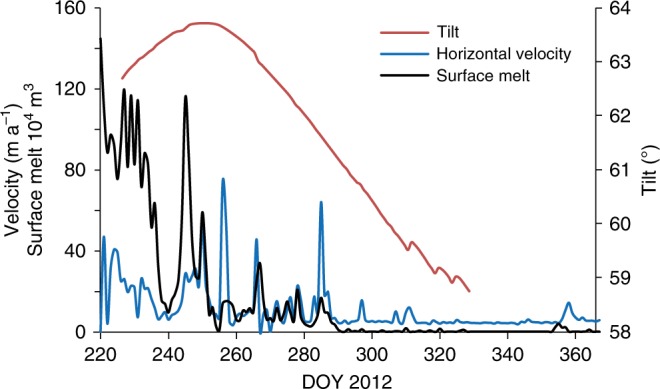
Graph showing data from 2012 to 2013; daily melt, mean horizontal velocity from the four differential global positioning stations (dGPS) and tilt change from the wireless Glacsweb subglacial probes

Water pressure, tilt changes, and case stress data from an in situ probe embedded in the till, alongside surface melt from 2008 to 2010 are shown in Fig. 3a, with the winter of 2008/2009 shown in Fig. 3b. Probe tilt, surface melt and horizontal velocity data from 2012 to 2013 are shown in Fig. 4. We use our horizontal velocity data to show sliding and the tilt data to indicate till deformation.

The glacier overlies grey basalts and a fine grain till (mean grain size 53 µm), with evidence of subglacial deformation in the foreland, consisting of flutes and push moraines. The till is highly saturated at the glacier margin. Data from repeated 50 MHz ground penetrating radar (GPR) surveys (summer 2008, 2011, 2012) showed a low water content within the main body of the glacier (water content 0–0.5%), a thin debris-rich basal layer (1 m thick, water content 2%), and what appear to be braided subglacial channels accounting for ~6% of the area of the bed (with a typical width in summer of 0.5–15 m, mean 3 m)^44^. Previous research also indicated that icequakes were being generated from the glacier bed, and that there was a positive relationship between daily melt water production and the number of seismic events^46^.

### Melt season behaviour

**Fig. 5:**
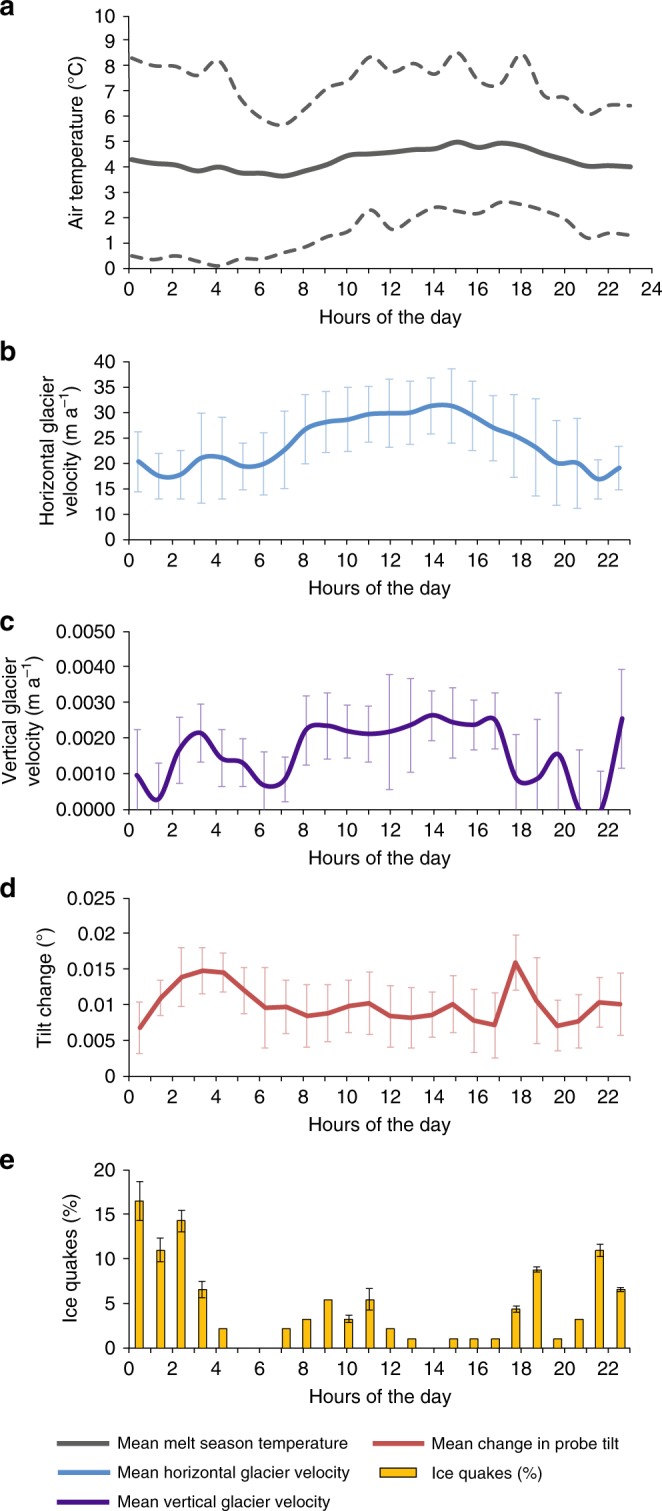
Graphs showing mean melt season diurnal data (2012): **a** air temperature (mean shown as a solid line, the 10% warmest and 10% coolest days are shown as a dashed line); **b** horizontal glacier velocity; **c** vertical glacier velocity; **d** probe tilt change; **e** Icequakes (plotted as an hourly percentage) (error bars are proportional to one standard deviation)

During the melt season (spring, summer and autumn) average daily temperatures were above zero at the field site, and there was significant melting over the glacier (typically day of year (DOY) 121–289). Air temperatures rose during the morning (from 07:00 to 12:00) to a high level in the afternoon (12:00–17:00), and then fell in the evening and overnight (17:00–07:00) (Fig. 5).

The daily glacier surface horizontal velocities were variable (Fig. 4) and although the mean daily velocities were higher during the elevated melt periods there is no direct relationship between the magnitude of the daily melt water input and the velocity magnitude. Diurnal patterns include an increase in horizontal and vertical velocity each day (Fig. 5). This speed-up started between 10:00 and 13:00, and the timing remained approximately the same throughout the melt season. The mean peak in horizontal velocity was 153% greater than the minimum velocity. On most days (76%) the peak in horizontal velocity occurred during the first 2 h of the afternoon high temperature period (so there was an approximate 2 h lag between melt water input and velocity response). Then velocities decreased whilst temperatures were still high. On most days (77%) there was a small secondary velocity rise (35% greater than the minimum velocity) overnight (between 23:00 and 6:00).

The vertical velocity pattern was very similar with a major rise each day during the speed-up, a decrease in the afternoon (whilst the air temperatures were still relatively high) and then a small rise overnight.

Changes in tilt show a diurnal pattern comprising low tilt changes during the velocity peak itself, followed by two periods of high tilt changes (in the afternoon during the velocity decline, and overnight, during the small velocity increase), a short period of low tilt change in the early morning, and finally an intermediate level of tilt change as the temperature warmed during the morning. The periods of high and intermediate tilt changes were accompanied by oscillatory changes. The icequakes follow a similar pattern, with very few occurring during the velocity peak, then numerous events during the afternoon fall in velocity and the small rise in overnight, followed by very few during the coolest part of the day (early morning) with increasing numbers as the temperatures rise (Fig. 5).

### Winter behaviour

During winter, where average temperatures are below zero and so on most days there is no melting, there was a base winter velocity together with distinct speed-up events during high temperature/melt water events (typically >2 °C at the Base Station or melt of 100,694 m^3^) (Figs 3 and 4). During the speed-up events, glacier surface horizontal velocities were up to 500% faster than the base level winter horizontal velocity, and lasted between 1 and 4 days. These events were also accompanied by vertical uplift (bed separation). At the same time, the horizontal flow direction underwent an abrupt 90^o^ displacement of 0.13 m to the NE (towards the centreline of the glacier), and then continued to flow in the same direction as before (towards the SW) until the next event.

**Fig. 6:**
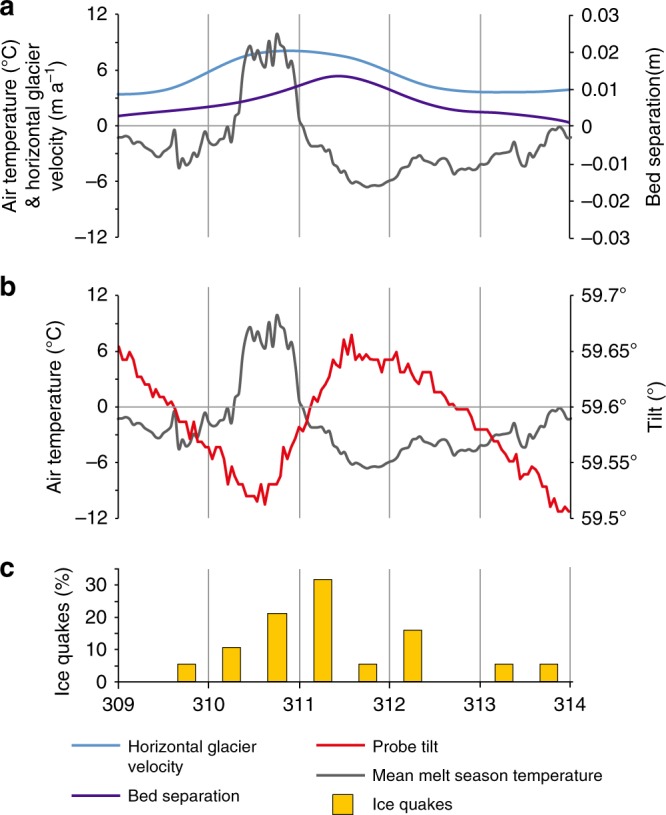
Graphs showing typical winter multi-day melt event (day of year 309–314, 2012): **a** hourly air temperature, daily horizontal glacier velocity and daily bed separation (see text for details); **b** hourly air temperature and hourly probe tilt; **c** icequakes (plotted as percentage for each 12 h period over the 5 day cycle)

The velocity began to increase on the same day as the meltwater input throughout the winter, but the timing of the peak in velocity (after the melt water input) varied between a few hours (38% of the events), to 1 day (50% of the events) to 3 days (12% of the events) (Fig. 6). The days with an immediate velocity peak mostly occurred during the early winter (DOY 290–309), the one day lag to the velocity peak occurred slightly later (DOY 310–324), whilst the melt event with the longest response to peak velocity occurred towards mid-winter (DOY 355–359) associated with a large melt event after a period of sustained low temperatures (lasting 25 days).

During the winter, water pressure followed a cyclical pattern (Fig. 3). On each melt event (over the temperature/melt threshold), there was a sharp water pressure decline followed by a slow rise in water pressure on subsequent days until the next melt event. These water pressure changes were accompanied by a cyclical pattern in tilt change. On the day of the melt event there was a small antithetic change in tilt. This is succeeded by a large synthetic movement during the following day(s). When the water pressure was low, the tilt change was also zero or very low, and on subsequent days both water pressure and tilt change increased. The water pressure threshold for the inception of tilt movement was not constant throughout the cycles (Table 1). For each of the first three cycles (DOY 290–302, 303–311, 312–242), the threshold in water pressure, and numbers of days to reach it, declined. During the fourth cycle (DOY 343–19), tilt movement occurred after a higher water pressure threshold.

**Table 1:** The water pressure threshold for the inception of tilt movement for Probe 21 for each water pressure cycle (2009/2010)

Water pressure cycles	DOY	Number of days with low or no tilt	Water pressure at inception of tilt movement (m w.e. %ht)	Applied force at inception of tilt movement (N)	Effective pressure at inception of tilt movement (kPa)
1	290–301	9	76.41	282.78	68.87
2	302–312	4	73.27	284.35	86.12
3	313–342	2	72.83	285.90	88.54
4	343–020	7	76.06	285.90	70.80

Deformation in the till occurred throughout the winter (recorded by change in tilt), both during the negative and positive degree days (Fig. 4). However, antithetic behaviour was only recorded on days with a positive degree day. During 2012, there were three antithetical events that all began around midday during the melt events on DOY 310, 317 and 323 and lasted an average of 16 h (Figs 4 and 6) with smaller oscillatory events occurring during the temperature increase, peak and decline. During winter icequakes also occurred. The vast majority of events (84%) occurred during the positive degree days. On these days, 16% occurred before, 21% during and 63% after the temperature peak during a typical 5 day cycle (Fig. 6).

### Effective pressure, till strength and icequakes

**Fig. 7:**
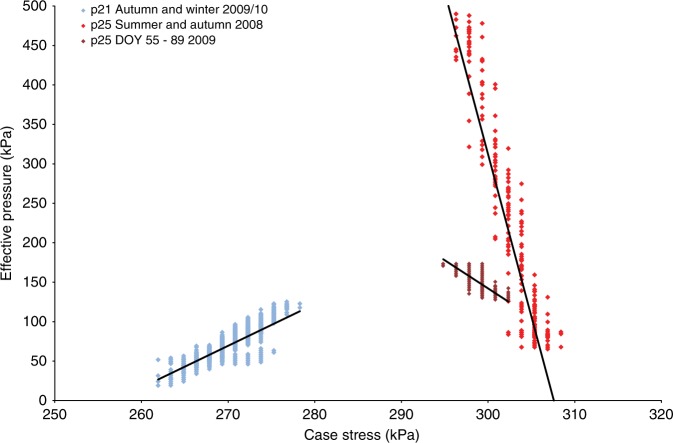
Graph showing the relationship between case strain (till strength) and water pressure for the subglacial probes (2008–2010 data)

The relationship between subglacial effective pressure (ice-overburden pressure minus the water pressure) and till strength can reflect changes in till behaviour. We use case stress as a proxy for till strength^37^ in a similar way to previous studies using ploughmeters^41,42^. We also find both positive (probe 21 winter 2009/2010) and negative (probe 25) relationships (Fig. 7)^37,41,42^. The former reflects a strengthening of the sediment with increasing effective pressure^22^, whilst the latter reflects weakening^26^.

It has been argued that there is a relationship between effective pressure and icequakes^17,18^. We can calculate this value from our data from Skálafellsjökull (see Methods), which predicts a very low effective pressure (above which icequakes can occur) (~1 kPa for melt season and winter), equivalent to below 98% flotation pressure.

## Discussion

We suggest our results demonstrate stick-slip motion, because they have a distinct pattern of water pressure, velocity, till deformation and icequake behaviour. We compare and contrast melt season and winter styles of stick-slip motion, and relate these to changes in effective pressure and how the subglacial hydrological systems responds to varying melt inputs. We demonstrate how these glacial processes effect till sedimentation, and propose this behaviour is typical of bedded glaciers, which are sensitive to surface driven melt.

**Fig. 8:**
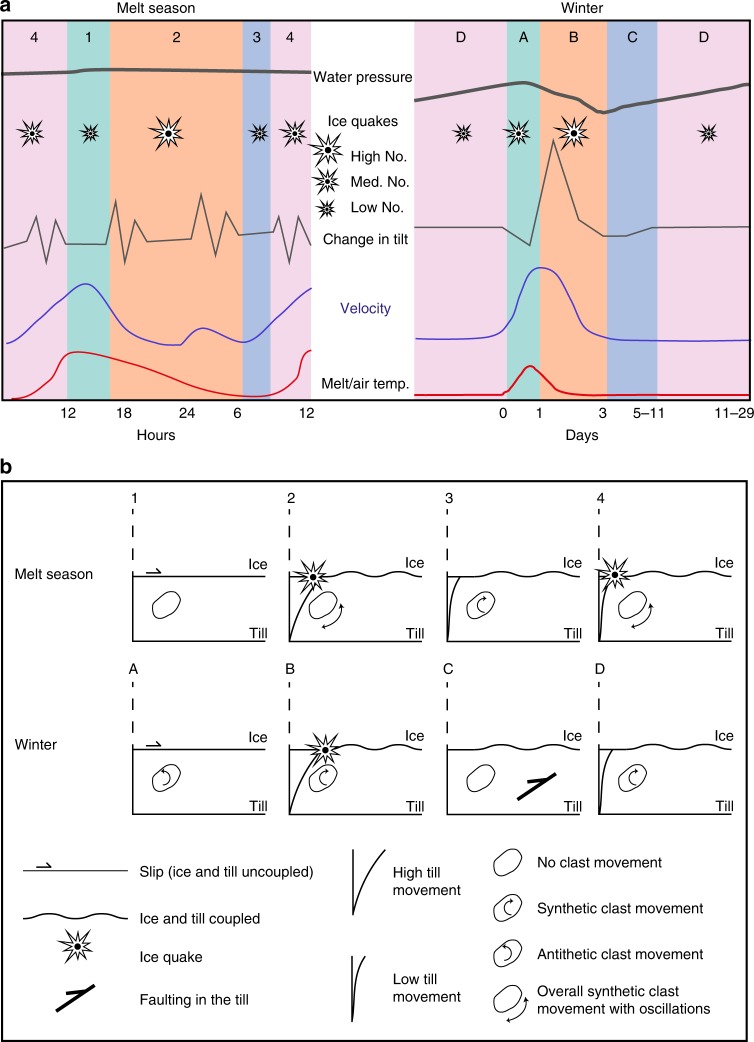
Schematic diagram to demonstrate the four stages of stick-slip behaviour in the different seasons. **a** This shows a graphical representation of water pressure, icequakes, tilt change direction, glacier horizontal velocity and meltwater input during a melt season (diurnal) and winter (multi-day) cycle. Melt season stages: 1—sliding, 2—reconnection, 3—minimum deformation and 4—reactivation; winter stages: A—sliding, B—reconnection, C—stick and D—reactivation. Icequakes represented as percentage of the total over the cycle: low number =<15%, medium number = 15–60%, high number = >60%. Change in tilt represented as follows: up = synthetic movement, down = antithetic movement, zig-zag = oscillatory movement associated with overall synthetic movement (large = > 0.02° h^−1^; small = 0.01–0.02° h^−1^). **b** This shows a diagrammatic representation of the subglacial environment (ice, till and ice/sediment interface) of the four stages during the melt season and winter

The two distinct seasonal styles of behaviour can be divided into four stages (sliding, reconnection, minimum deformation/stick and reactivation) (Fig. 8). The melt season was characterised by a diurnal cycle. Temperatures and surface melt increased each morning until a threshold was passed, which resulted in basal sliding, accompanied by little change in tilt and few icequakes (Stage 1, sliding, mean length 4.2 h).

As the velocities decreased in the afternoon there was reconnection with the bed (Stage 2, reconnection, mean length 12.3 h), which was accompanied by high tilt movement and a significant number of icequakes. This was indicative of till deformation. Once the sliding was activated (during Stage 1), continued supplies of melt water (during the afternoon) had little effect, as have been reported elsewhere^12,47^. However, once melt water levels fell below a certain threshold (as temperatures cooled overnight), melt water and velocity became directly related, leading to the short term velocity rise (mean length 4.5 h).

This continued until air temperatures reached their lowest at during early morning. During this time glacier velocities were also low, and there was relatively low tilt movement and a decreasing number of icequakes (Stage 3, minimum deformation, mean length 4 h). As temperatures rose each morning, this enabled increased melt water to reach the bed, allowing the glacier to advance; this was associated with increased till deformation and associated icequakes (Stage 4, reactivation, mean length 3.5 h).

The rheological relationships during the melt season were complex. Over a long time scale (the whole summer of 2009) there was a positive relationship between water pressure and till strength (Fig. 7). However, over a short time scale (diurnal cycle), there was no significant relationship between water pressure and till strength as water pressures were almost always high.

Winter was characterised by multi-day cyclical behaviour. On the positive degree days, supraglacially generated melt water rapidly flowed to the base of the glacier (Stage A, sliding, mean length 1 day), which resulted in basal sliding (up to 500% faster than the base-level winter velocity), which was accompanied by antithetic motion due to unloading and reorganization of the grains^41,48–50^. This also resulted in bed separation and an abrupt displacement of the glacier flow direction. During glacier deceleration, the glacier reconnected with the bed (Stage B, reconnection, mean length 2.25 days). At the same time, water pressure dramatically decreased, accompanied by a return to synthetic tilt movement, combined with dilation and grain reorientation (dilation strengthening) and deformation. This continued until water pressure had fallen below a threshold for till deformation (Stage C, stick, mean length 6 days). After this, water pressures rose (above the threshold for deformation), and the till deformation resumed (Stage D, reactivation, mean length 13.5 days).

The critical water pressure level for failure decreased for the first three short cycles (Table 1). This was similar to other reported results^3^ that showed that the thickness of the deforming layer increased after a series of repeated similar cycles of slick-slip behaviour, i.e. the till became easier to deform. However, during the fourth cycle, the critical water pressure for failure increased. This may reflect the system becoming reset, after the extreme unloading and reorganization of the grains during the large water pressure decline DOY 344–347 (Fig. 3b).

The main differences between the winter and melt season patterns were threefold. Firstly, during winter there was a long (mean length 21 days) period during which the glacier was coupled to its bed and so strain energy was being accumulated (Stages B, C and D). This strain was released during Stage A, resulting in the dramatic antithetic motion. Secondly, during winter there was the dramatic decrease in water pressure, which resulted in a stronger reconnection and associated synthetic motion (Stage B). Finally, in winter there was a definite stick phase (Stage C), whilst during the melt season water pressures in the till were so high that they never fell below a critical level for failure (Stage 3).

The rate-and-state friction model suggests that slip-stick cycles relate to an evolving basal shear strength that is proportional to effective pressure^18^. Our results are partly consistent with a model that suggests icequakes only occur below a critical water pressure level^17,18^. In winter the critical water pressure level was never crossed, and icequakes occurred throughout the cycle (even during the sliding phase), but were most frequent during glacier reconnection with the bed. However, during the melt season, there was no diurnal change in water pressure; however, the high water pressure conditions may have been ideal for icequakes during the morning (Stage 4) and evening (Stage 2), whilst the during the early morning (Stage 3) and the slip phase (Stage 1) there was insufficient loading for icequakes to occur.

We suggest the main contrast in styles relates to the relative amounts of melt water and how the subglacial hydrological system responds^51,52^. During high melt water events, the glacier hydrological system is unable to accommodate the melt water influx and the glacier rapidly slides forward. During the melt season this was due to the regular diurnal temperature rise, and the lag between melt water inputs and velocity increase was typically 2 h, but in winter it was the result of irregular warm events, with response times ranging from a few hours to 3 days. During the melt season there was a continual supply of water from the glacier surface to the bed and the subglacial hydrological system could relatively easily adapt to any melt water increase. In contrast, during winter, the rare melt water inputs overwhelmed the subglacial hydrological system. This resulted in immediate velocity increases, but the length of time to peak velocity varied. At the beginning of winter, the velocity peak was almost immediate, whilst later in the winter there was an increasing lag time between the melt event and the maximum velocity response. This must reflect the gradual closing of the melt water pathways (both englacial and subglacial) as the winter progressed. Throughout the winter, the sliding was accompanied by relaxation in the till (antithetic behaviour), significant vertical (bed separation) and lateral ice movement and a dramatic drop in water pressure which caused a reorganization of the drainage system.

These insights into the two types of stick-slip motion have implications for till formation. Prior to the 1970’s it was assumed that most till was deposited by lodgement (due to frictional retardation at the ice-sediment interface)^38^. However, in recent years it has been acknowledged that the formation of subglacial till is extremely dynamic and controlled by changes in effective pressure, grain size, relative sediment strength and hydrology with an emphasis on the importance of deformation^37,38,53,54^.

In this study, we were able to quantify changes in subglacial depositional processes over a season and show how the till beneath Skálafellsjökull can be deformed. We have shown that the till can be deformed all year, but undergoes different styles of deformation (Fig. 8). The different stages of the stick-slip deformation cycle may be associated with different forms of deposition.

The sliding phase may be associated with lodging. That is, clasts held within the ice are dragged through the till, and deposited due to friction. Sedimentary evidence for lodging in the foreland may be represented by the core clasts of flutes. In contrast when the glacier was coupled with the till, subglacial deformation occurred and produced a deformation till (as well as the body of the flute) with a dominance of ductile microstructures, and evidence for grain percussion and abrasion^55^.

During the melt season, clasts within the ice and the deforming layer experience diurnal cycles. Lodging associated with basal sliding comprises 18% of the day, whilst deformation occurs during the remainder (reconnection 51%, minimum deformation 17% and reactivation 14% of the day). The periods of glacier acceleration and reconnection (during periods of maximum icequakes) may also be associated with grain/clast fracture. This demonstrates the typical disruptions to a constant strain pattern associated with subglacial deformation, which creates the complex patterns obtained from the fabric and deformation structures typically found in subglacial till^56,57^.

During the winter, the till will undergo multi-day changes (mean 22.75 days). Antithetic motion in till and possible lodging at the ice-sediment interface (associated with basal sliding) occurred during 6% of the cycle. Deformation in the till occurred during the acceleration (37%) and reconnection (16% of the cycle) but at a slower rate than the melt season. During the remainder of the time, there was no ductile deformation, although there may be brittle deformation (till faulting).

We can make a quantitative estimate of the amount of time each process may occur over a given year and its implication for overall sedimentation (Table 2). Basal sliding (lodgement) accounts for ~10%, ductile deformation for 70% and stick (brittle deformation) for 20% of the whole year. Over the whole year, these processes are superimposed on one another to produce the resultant till. Clasts that were originally lodged during sliding may be rotated during deformation. The effect of this is a complex till with elements of lodging, ductile and brittle deformation, but with an overall response to melt water inputs, resulting in cyclical till deposition.

**Table 2:** Length of time for each stage throughout the seasons

Stage		Melt season %	Winter %	Whole year %
1/A—sliding		18	6	11
2/B—reconnection		51	11	29
3/C	minimum deformation	17	–	8
	stick	–	35	19
4/D—reactivation		14	49	33
Cycle length		24 h	22.75 days	365 days

We suggest Skálafellsjökull shows these distinct styles of stick-slip behaviour because it has a rapid englacial transfer rate, and a dynamic subglacial hydrological system (deforming bed dominated by thin water sheets, macroporosity and braided channels rather than large cavities and channels). Our results contribute to the debate as to whether increased melting leads to increased velocity^58^ or whether once an efficient subglacial drainage system develops, melt-induced acceleration of flow ceases^59,60^. Those glaciers which do not show melt-induced acceleration tend to be rigid bedded, and have an efficient channelized system and englacial storage. This observation is also supported by research at Hofsjökull^61^ where it was shown that the relationship between surface melt and basal sliding is a function of the mechanical properties of the deforming bed and the surface slope (i.e. low angle soft-bedded glaciers are sensitive to melt water production). These results have important implications because unconsolidated beds underlie many of the fast flowing ice streams of modern day Antarctic^14,16,18^ as well as the Quaternary ice sheets^2,32,38^, and we have been able to demonstrate a distinctive seasonal pattern of melt-driven stick-slip motion and the resultant till sedimentology associated with this glacial environment.

## Methods

### Environmental sensor network

This system comprised sensor nodes (Glacsweb probes and geophones) and base stations which are linked together by radio networks. Their data was transmitted from base stations via GPRS to a cloud server and hence to a sensor network server in the UK. Node data, along with differential GPS (dGPS) recordings and meteorological data, were sent once a day to a mains powered computer (16 km away), where it was forwarded to a web server in the UK^62^. The specific site location on the glacier was determined by the optimal depth at which the system can transmit data through the till and ice (50–80 m).

### Probes

Sensors housed within a probe (0.16 m long, axial ratio 2.9:1), measured water pressure, case stress, conductivity, tilt and temperature within the ice or till. Here we report only water pressure, case stress and tilt. These data were recorded every 15 min in 2012 and every hour in 2008–2010, and transmitted to a base station located on the glacier surface. These were deployed in the summers of 2008 and 2012, in a series of boreholes, which were drilled with a Kärcher HDS1000DE jet wash system. Once the boreholes were made, the glacier and till were examined using a custom made CCD colour video camera with infrared LED illumination.

In order to insert probes into the till, the boreholes were drilled to the base of the glacier and the presence of till checked with the video camera. If till was present it was hydraulically excavated by maintaining the jet at the bottom of the borehole for an extended period of time. The probes were then lowered into this space, enabling the till to subsequently close in around them. The measured depth of the probes (in the till) was estimated from video footage of the ice/till interface at 0.1–0.2 m beneath the glacier base.

Details and calibration of the sensors in the in situ probes were as follows. The water pressure sensors were calibrated against the measured water depths in the borehole immediately after probe deployment. The results were expressed as hydraulic head (m) (*h*_w_). The glacier thickness (*h*_i_) was determined from the GPR and GPS results. We calculate flotation pressure (%) (*ϕ*)^63^, assuming the density of ice (*p*_i_) is 917 kg m^−3^ and water (*p*_w_) is 1000 kg m^−3^.

1 $$\phi = \frac{{h_{\mathrm{w}}}}{{\left( {h_{\mathrm{i}}\rho _{\mathrm{i}}/\rho _{\mathrm{w}}} \right)}} \times 100$$

Case stress was defined as applied force against the probe, which was calibrated using an Instron 5560 tension/compression machine attached to a nitrogen cooled chamber, where the average chamber temperature was 1.3 °C. The Glacsweb probes measured tilt with two dual axis 180^o^ MEMS accelerometers. They were calibrated in the laboratory, and dip was calculated by trigonometry. Values of 0° x-tilt represent the probe standing vertically, and bearings reflect orientation, however, all readings here are represented as tilt changes.

In 2008, six probes were deployed, and four continued to send data after a day. Here we discuss the data from probe 21 as it had the most complete record. This probe was installed in the till beneath 58 m of ice and sent back data for 2 years with 45430 readings. The probes were designed so that if the data was not immediately accessed then it was stored for later retrieval. There were occasionally problems with communications between the probes and the base station which led to the probes filling their storage, resulting in some data gaps.

In 2012, four probes were deployed which all operated well. Here we discuss the results from the two subglacial probes (P31 and P32). P31 was beneath 69 m of ice, functioned for 105 days and sent back 78705 readings. P32 was at a similar depth (68 m), functioned for 74 days and sent back 67515 readings. Unfortunately only the temperature, probe deformation and tilt sensors functioned.

### Geophone nodes

Four geophone nodes were installed within boreholes, to avoid surface seismic noise, on a diamond grid, separated by a distance equivalent to the depth of the glacier. These comprise a custom built system, using an ARM® Cortex®-M3 processor (Energy Micro EFM32G880F128) with a low power design, running on battery power^46^. The geophone nodes continually sampled the output of three orthogonal geophones (28 Hz Geospace GS-20DH geophones encapsulated in resin inside a 70 mm diameter polycarbonate tube). Only data from seismic events are stored, held temporarily on a micro-SD card until they are retrieved by the base station. A 25 dB amplifier was used to provide sufficient signal. A bandpass pre-filter of 0.5–234 Hz was used, and a sampling rate of 512 Hz.

Due to the prototype nature of the enclosures and electronics only one geophone (ID 62) functioned for more than 1 day. However, this operated for 25 days in 2012 from DOY 270 to 295 in 2012. The signal was similar on all three planes. During this time 180 events were recorded^46^.

### dGPS

A dGPS system was used to map the glacier margin, boreholes, base station and radar grids. In order to measure surface velocities, a Topcon dGPS was used (2008–2012), with an additional four dual frequency Leica dGPS stations installed on the glacier in the study area, with a local base station on the moraine (2012–2013). These measured at a 15 s sampling rate continuously during the summer and 2 h per day during the winter. The GPS data was processed using TRACK (v. 1.24), the kinematic software package developed by Massachusetts Institute of Technology (MIT) (http://www.unavco.org/, http://geoweb.mit.edu/~tah/track_example/).

Both horizontal and vertical velocities were examined. The vertical motion of the surface ice is the sum of the downward vertical component of mean bed-parallel motion, thinning or thickening of ice associated with ice strain^64^, and bed separation^5^ and any till volume changes^65^ (Δz):

2 $$\Delta {\mathrm{z}} = - {\mathrm{\alpha }}_{\mathrm{v}}{\mathrm{L}}\Delta {\mathrm{\sigma }}$$

where Δ*σ* is the change in effective pressure, *L* the till thickness and *α*_v_ till compressibility (10^–8^ Pa^−1^).

### Weather data and daily melt

Weather data were obtained from a meteorological station sited on the base station and, during periods of failure, from a transfer function applied to data from the neighbouring Icelandic meteorological station at Höfn. Daily melt was calculated by the positive degree day algorithm^66^ using degree day factors for Satujökull, Iceland^67^, 5.6 mm d^−1^ °C^−1^ for snow and 7.7 mm d^−1^ °C^−1^ for ice. The elevation of the snow/ice boundary was inferred from MODIS albedo data, using a threshold of 0.45, on a 30 × 30 m grid ASTER DEM.

### Remote sensing glacier velocity

Velocity data was generated using the intensity tracking algorithm within the European Space Agency (ESA) Sentinel Application Platform (SNAP). Intensity tracking is less precise than interferometry but given the high temporal correlation of glacier surfaces, is much more robust^68^. The two TerraSAR-X SLCs were calibrated and co-registered together using a DEM-assisted co-registration using a digital elevation model (DEM) derived from airborne LiDAR data provided at 5 m resolution from the Icelandic National Land Survey. Velocities were then calculated using cross-correlation with a 5 × 5 moving window and a search distance of 64 pixels. Any displacements that had a cross-correlation threshold of 0.01 were removed, and the displacements were averaged to a 5 × 5 mean grid and converted to ground range.

### Critical effective pressure for icequakes

We have reported a method^17^ to calculate the critical effective pressure ($\bar \sigma$) for icequakes:

3 $$\begin{array}{*{20}{c}} {\bar \sigma = \frac{{\eta V_{\mathrm{s}}}}{{b - a}} } \end{array}$$

here η = radiation damping parameter^69^

4 $$\begin{array}{*{20}{c}} {\eta = \left( {\frac{1}{{z_i}} + \frac{1}{{z_2}}} \right)^{ - 1}} \end{array}$$

*z*_i_ and *z*_b_ are the shear wave impedances of the ice and bed, *V*_s_ = surface velocity, *a* and *b* represent frictional parameters. We use the following values *z*_i_ = 2000 m s^−1 ^^17^, *z*_b_ = 150 m s^−1 ^^32^ (from nearby Breiđamerkurjökull), *a* = 0.010, *b* = 0.015^17,18,21,40^, *V*_s_ *=* 30 m a^−1^ during the melt season and 10 m a^−1^ during winter. This method cannot applied where the water pressure exceeds the overburden pressure.

## Data Availability

Data is available at www.glacsweb.org and from JKH (jhart@soton.ac.uk).
